# Rapid determination of levels of the main constituents in e-liquids by near infrared spectroscopy

**DOI:** 10.1038/s41598-023-40422-z

**Published:** 2023-08-19

**Authors:** Anaïs R. F. Hoffmann, Jana Jeffery, Paul Dallin, John Andrews, Michał Brokl

**Affiliations:** 1 B.A.T. (Investments) Limited, Regents Park Road, Millbrook, Southampton, SO15 8TL UK; 2 Clairet Scientific Limited, 17/18 Scirocco Close, Moulton Park Industrial Estate, Northampton, NN3 6AP UK

**Keywords:** Chemistry, Analytical chemistry, Infrared spectroscopy, Mathematics and computing, Statistics

## Abstract

Use of e-cigarettes is increasing, alongside an expanding variety of devices and e-liquids. To match this growth and in line with the expanding legal and regulatory requirements applicable to manufacturers of e-cigarettes (e.g. disclosure of list of ingredients and quantities thereof in a product), rapid methods for determining levels of the main e-liquid constituents—namely, propylene glycol (PG), vegetable glycerol (VG), water and nicotine—are needed. We have assessed the ability of near infrared (NIR) spectroscopy, coupled with partial least squares (PLS) regression, to predict the levels of these constituents in e-liquid formulations. Using NIR spectral data from a large set of reference e-liquids incorporating working concentration ranges, flavourings, and other ingredients, linear calibration models were established for PG, VG, water and nicotine (predicted vs theoretical values, all *R*^2^ > 0.995). The performance of these models was then evaluated on commercial e-liquids using NIR and compared to results obtained by gas chromatography (GC). A strong correlation was observed between NIR-predicted values and measured values for PG, VG and nicotine (all *R*^2^ > 0.955). There was less consistency between predicted and GC measured values for water due to the relatively high limit of quantification (LOQ) of the GC method (2.6% w/w) versus the e-liquid content (0–18% w/w). The LOQ of the NIR method for water was 0.6% w/w, suggesting that NIR may be a more accurate method than GC to predict water concentration in e-liquids, especially at low levels (< 2.6% w/w). Collectively, although limitations of the technique have been identified, specifically for e-liquids containing compounds that might interfere with the set calibrations, our findings suggest that NIR combined with PLS regression is a suitable tool for rapid, simultaneous and high-throughput measurement of PG, VG, water and nicotine levels in most commercial e-liquids.

## Introduction

Awareness and use of electronic cigarettes (e-cigarettes) have grown significantly over the past decade. Although e-cigarettes may differ considerably in shape, size, power and airflow, they all consist of a battery, inhalation mechanism and e-liquid, supplied either in a pre-filled cartridge (closed system) or in a tank that can be refilled by the user (open system). A large selection of e-liquids is available, but formulations typically contain propylene glycol (PG), vegetable glycerol (VG) and water in varying combinations and may also contain flavourings and/or an active compound such as nicotine.

In line with the continual expansion of product availability, regulations for e-cigarettes and liquids, especially around their composition (nicotine, flavourings) have been implemented and are evolving with the current market dynamics ^1–4^. Specifically, many of the legal and regulatory requirements inherent to tobacco product manufacturing have been expanded to electronic nicotine delivery systems and are therefore applicable to manufacturers of e-cigarettes ^5^. Regarding e-liquids, manufacturers must supply a list of all ingredients, and quantities thereof, contained in the e-liquid ^6–8^. However, a recent study of commercial e-cigarettes in Australia reported a discrepancy between the claimed and measured PG and VG concentrations ^9^, which demonstrates the need to monitor e-liquid content.

To date, only a few studies have described the analysis of key e-liquid ingredients, with methods mostly based on gas chromatography–mass spectrometry (GC–MS) ^9–12^. However, sample preparation and analysis by GC, or other conventional methods (e.g., nuclear magnetic resonance) is often lengthy. For the rapid determination of PG, VG, water and nicotine in e-liquids (e.g., for quality control purposes), an approach that reduces sample processing time and analytical time, allows high sample throughput and provides acceptable accuracy would be highly desirable.

Near infrared spectroscopy (NIR) has applications in industrial quality control and process monitoring. It is commonly used in the analysis of pharmaceuticals, medicines, cosmetics and foodstuffs ^13,14^, including to test for authenticity. NIR methodology is rapid (spectrum acquisition in minutes) and non-destructive, needs little to no sample preparation and, subject to spectral deconvolution, allows the simultaneous measurement of multiple analytes. NIR spectra are dominated by absorbances from CH, NH and particularly OH species. The major components of e-liquids (PG, VG, water and nicotine) contain many of these species and so have strong NIR absorbances, making this technique potentially suitable for the analysis of e-liquids ^15^. NIR spectra are often complex because the absorption bands are naturally very broad and overlap; therefore, simple (univariate) models based on peak height/area are rarely applicable. However, it may be possible to predict the levels of PG, VG, nicotine and water in e-liquids by deriving calibration models, referred to as “chemometric models”, for each analyte based on multivariate methods such as projection to latent structures (also known as partial least squares or PLS).

PLS is particularly useful when applied to the analysis of complex, and often noisy, data sets (such as spectra) and their relationship to key chemical parameters, such as analyte levels ^16^. Once a PLS model is established in NIR, the prediction of analyte concentrations in an unknown sample is possible based on its spectrum ^17^. Improvements in computing power have facilitated the application of such PLS models allowing the rapid and automated processing of spectral data. Results are therefore obtained in near real-time, which allows decisions to be made promptly after the analysis of a sample. NIR analysis may offer the opportunity to simultaneously determine all major components of an e-liquid from one spectrum obtained from one instrument within minutes. Once established, the calibration can be transferred between compatible instruments (same manufacturer, software), reducing the need for additional calibration.

The aim of the present study was therefore to examine whether the application of NIR spectroscopy combined with PLS regression to the simultaneous and high-throughput measurement of PG, VG, water and nicotine levels in e-liquids is an effective alternative to other classical analytical methods. The accuracy of a chemometric model for the prediction of analyte concentrations in an unknown sample is dependent on the number of variables used to establish the calibration ^18^. Therefore, NIR spectral data were acquired from a comprehensive range of e-liquids (i.e.*,* analyte concentration ranges, inclusion of other ingredients, flavouring compounds etc.) to establish the calibration database. The performance of the established models was subsequently assessed by analysing up to 49 commercial e-liquids by NIR and comparison to results achieved by GC.

## Results

### Selection of NIR regions suitable for e-liquid analysis

**Figure 1:**
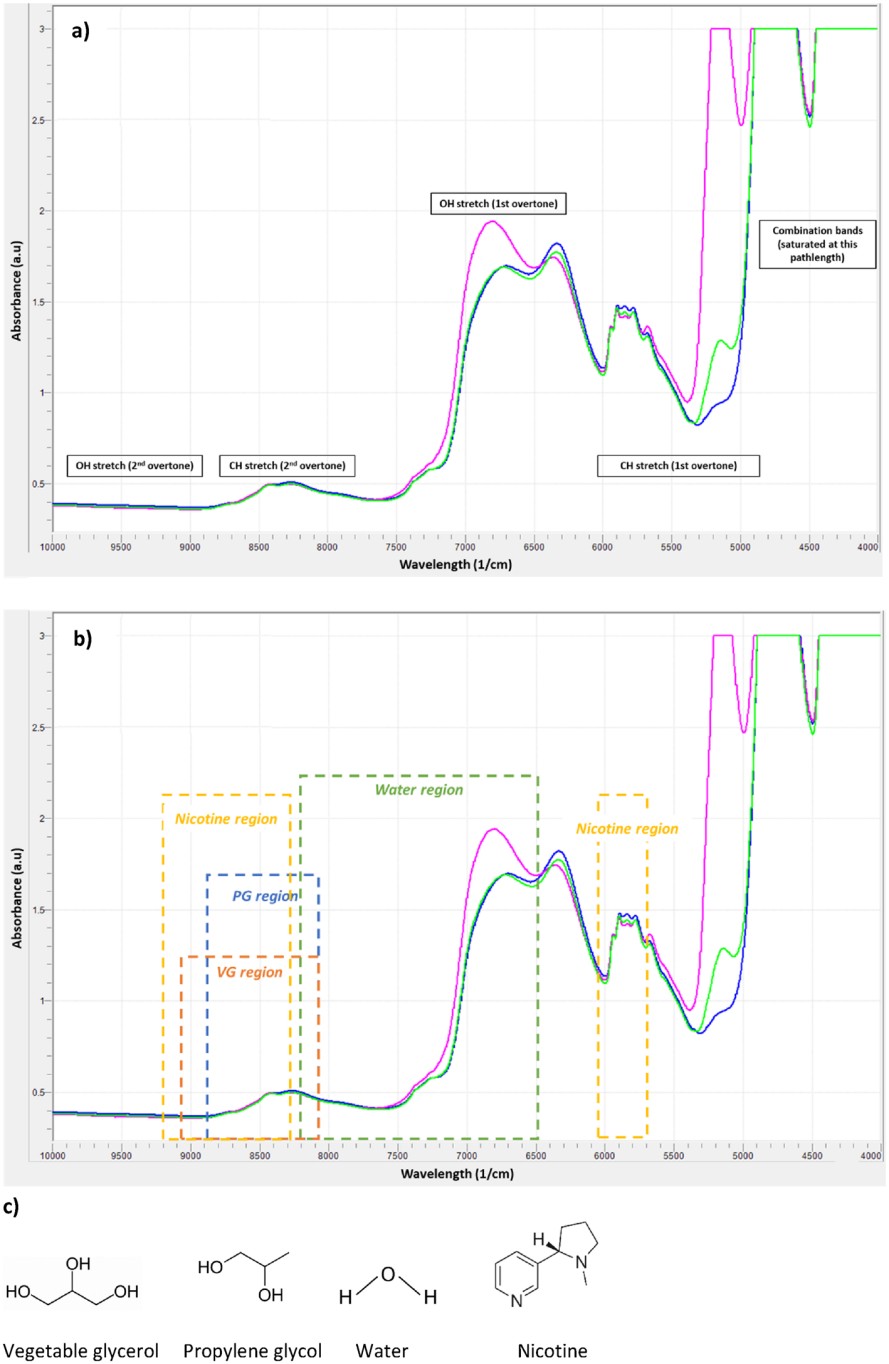
Representative NIR spectra of e-liquids and structures of the e-liquid constituents investigated in this study. (**a**) NIR spectra of e-liquids containing nicotine. Absorbance bands characteristic to the main constituents (PG, VG, water, nicotine) are observed at 4900, 5900, 6300, 6800, 7200, 8400 and 10,000 cm^−1^. (**b**) Integration regions used for PG (blue), VG (orange), nicotine (yellow) and water (green) calibrations. (**c**) Molecular structures of key constituents of e-liquids.

For application of NIR spectroscopy to the analysis of e-liquids, it was necessary to establish calibration models for the constituents of interest. The accuracy of these calibration models is based on the selection of wavelengths in the NIR spectrum that are relevant to the target analyte and display the most variation during pre-processing of the spectra. The chemical structures of PG, VG, nicotine and water all contain functional groups relevant to NIR (C–H, O–H) that show characteristic absorption in similar regions of the NIR spectrum (Fig. 1). This leads to multiple overlaps, especially in the 5800–8600 cm^−1^ region, which corresponds to the first and second overtone of the C–H stretching vibration (5700 and 8300 cm^−1^, respectively), and the first overtone of the O–H stretching band (6900 cm^−1^) ^13,19^ (Fig. 1 and Table 1). Specific regions of interest outside this area were observed in the spectra. For example, a distinctive sharp peak was observed for water at around 5000 cm^−1^ (O–H combination); however, the strong absorbance of this molecule in this NIR region led to detector saturation, which hindered quantification of the compound. Therefore, to discriminate among compounds and ensure the accuracy of each model for the selected analyte, the NIR regions for integration in the calibration models were selected in accordance with the following criteria:

- relevance to the target analyte;
- spectral variability observed during pre-processing of the spectra;
- selectivity of the wavelength (region displaying the least overlap with other analytes);
- intensity of the absorbance at the selected wavelength.

**Table 1:** NIR peaks and region of interest per analyte—the focussed regions of interest used for the PLS models are detailed in Table 2.

Component	NIR regions of interest (cm^−1^)
Vegetable glycerol	5843–6325
Propylene glycol	7262–7376, 8440
Nicotine	4403, 5770–5963
Water	4400, 5150, 6940, 8600, 10300

The detailed regions of interest used for the PLS models are detailed in Table 2.

**Table 2:** PLS model parameters and statistics for the calibration of PG, VG, water and nicotine concentrations in e-liquid.

Analyte	Conc. (% w/w)	No. of samples		Region (cm^−1^)	No. of factors	Standard error		*R*^2^
		Calibration	Validation			Calibration	Expanded	
PG	30–70	125	11	8038–8856	2	0.92	1.84	0.995
VG	18–65	123	14	7996–9041	2	0.94	1.88	0.995
Water	0–18	124	12	6441–8212	4	0.31	0.62	0.997
Nicotine	0–6	221	47	5701–6067; 8274–9192	9	0.11	0.22	0.995

*PG* propylene glycol, *VG* vegetable glycerol, *PLS* partial least squares.

### Calibration and validation

To establish calibration models for the main e-liquid constituents, NIR spectra for reference sets of e-liquids formulated in accordance with defined criteria, such as working concentration range for each analyte and chemical composition, were obtained. The main components of e-liquids are PG, VG, water and an active ingredient such as nicotine, but many commercial formulations are more complex, containing compounds such as flavourings, acids and/or other functional agents. Because these components may interfere with the calibration model’s ability to predict analyte levels, the sets of e-liquids used for calibration incorporated similar variability (e.g., flavourings, acids).

After data acquisition, the sample spectra were pre-processed using a second order Norris gap algorithm with a gap length that was adapted for each analyte to optimise the fit of the algorithm. This processing allowed to isolate the regions where the greatest variability across samples could be observed. A PLS regression was subsequently used to construct models to predict the analyte concentrations in the reference samples. To ensure the accuracy of the calibrations, a specific number of factors have been set for the models of each analyte. The suggested number of factors to build the model was initially determined by the software’s calibration wizard, which generates a factor analysis, after data input (Supplementary Tables 1–4). This number was subsequently adjusted with the use of a scree plot, generated by the software to optimise the predictive model ^20^.

**Figure 2:**
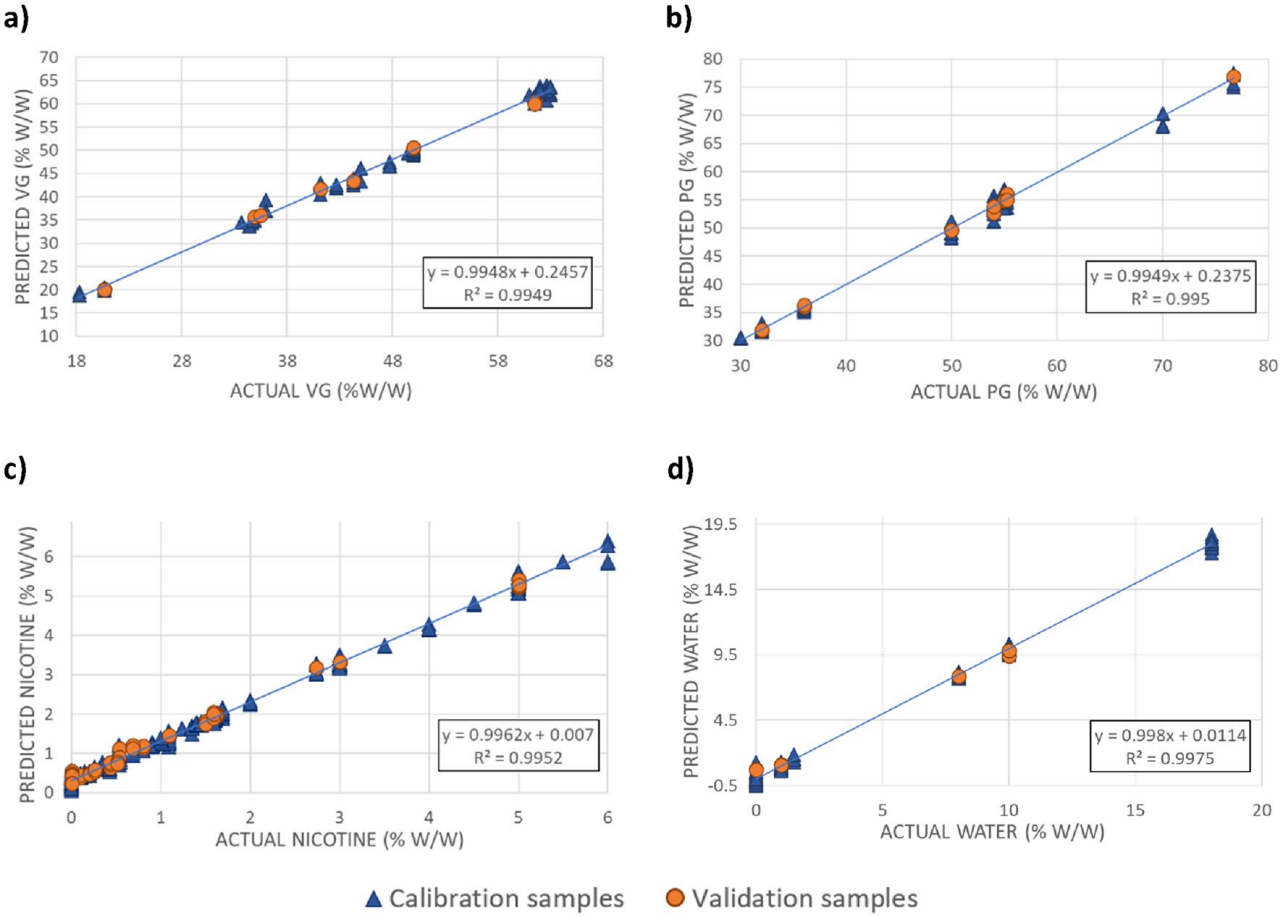
NIR calibration curves for (**a**) vegetable glycerol (VG), (**b**) propylene glycol (PG), (**c**) nicotine, and (**d**) water.

The parameters for each model and statistical outputs are summarised in Table 2. Comparison of the predicted values with the reference (theoretical) values used for the calibration allowed observation of both the quality of the PLS model and the correlation between the two values. A strong correlation between the values predicted from the spectral data and the reference values was observed for PG, VG and water (*R*^2^ > 0.995; Fig. 2), supported by fewer factors (2, 2 and 4 factors, respectively). A potential limitation of the NIR method is its sensitivity to analytes present at lower concentrations (e.g. flavourings, colourings). Here, the low levels (< 5%) of active compounds (i.e., nicotine) resulted in models with a higher number of factors (9), suggesting that the calibration models established for nicotine might not be as stable as those established for PG, VG and water.

### Assessment of NIR method

**Figure 3:**
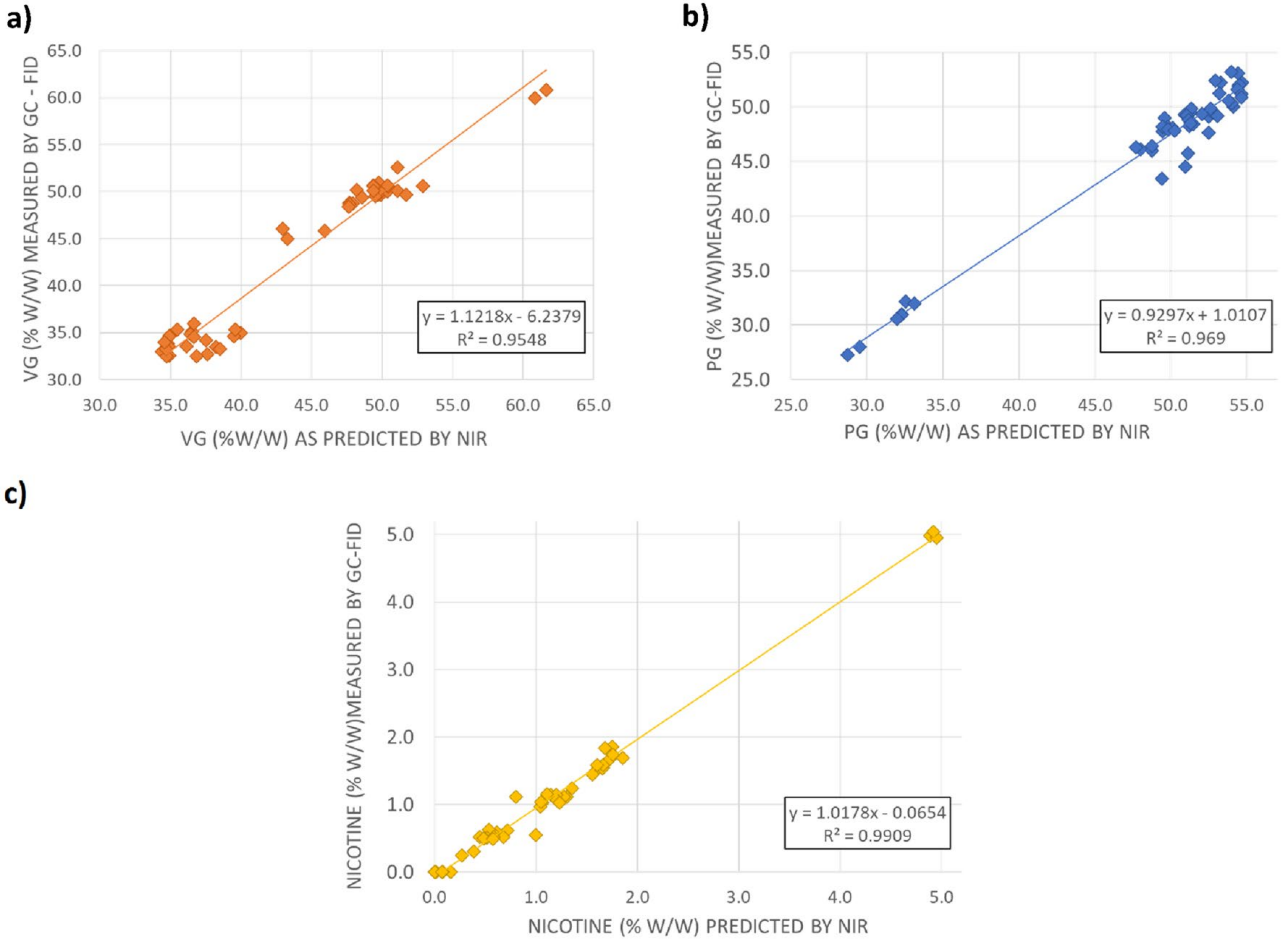
Comparison between predicted NIR values and GC measured values for (**a**) VG, (**b**) PG and (**c**) nicotine.

To assess the performance of the PLS models, 49 commercial e-liquid samples were analysed for PG, VG, water and nicotine by NIR spectroscopy and compared to results obtained by GC-FID (Flame Ionisation Detector) or GC-TCD (Thermal Conductivity Detector). The predicted NIR values were plotted against the measured GC data. Comparable results between the NIR predicted and measured values for PG, VG and nicotine were observed (all *R*^2^ > 0.95; Fig. 3 and Table 3, Supplementary Information—Tables 10, 11 and 12).

**Table 3:** Performance of the NIR method for PG, VG and nicotine analysis.

Analyte	Slope^a^	*R*^2^	Number of samples showing recovery	
			Within 90–110%	Outside 90–110%
PG	0.930	0.969	47 (96%)	2 (4%)
VG	1.122	0.955	42 (86%)	7 (14%)
Nicotine	1.018	0.991	34 (69%)	15 (31%)

^a^Slope calculated between NIR predicted values and GC measured values.

While the comparison between nicotine values obtained by GC-FID and NIR spectroscopy analysis showed a high correlation (*R*^2^ = 0.991; Fig. 3), a significant proportion of NIR samples (31%) had recoveries outside the defined acceptance window (90–110%). This is due to the size of the Standard Error of Prediction (SEP = 0.2% w/w) relative to the low working concentration range (0–6% w/w nicotine; most values < 2%). A direct comparison of the GC and NIR values for nicotine, taking into account the SEP for NIR measurements, gave a better representation of the data and showed that the predicted and measured values were consistent (Supplementary Fig. 1). In addition, the presence of minor compounds such as flavour ingredients may affect the prediction of nicotine level. Despite the overall consistency between the measured and predicted values, discrepancies were observed for some samples with the NIR value outside the prediction window (Sample 35, Supplementary Fig. 1). This observation suggests that some compounds present in e-liquids interfere with the measurement of nicotine by NIR spectroscopy.

**Figure 4:**
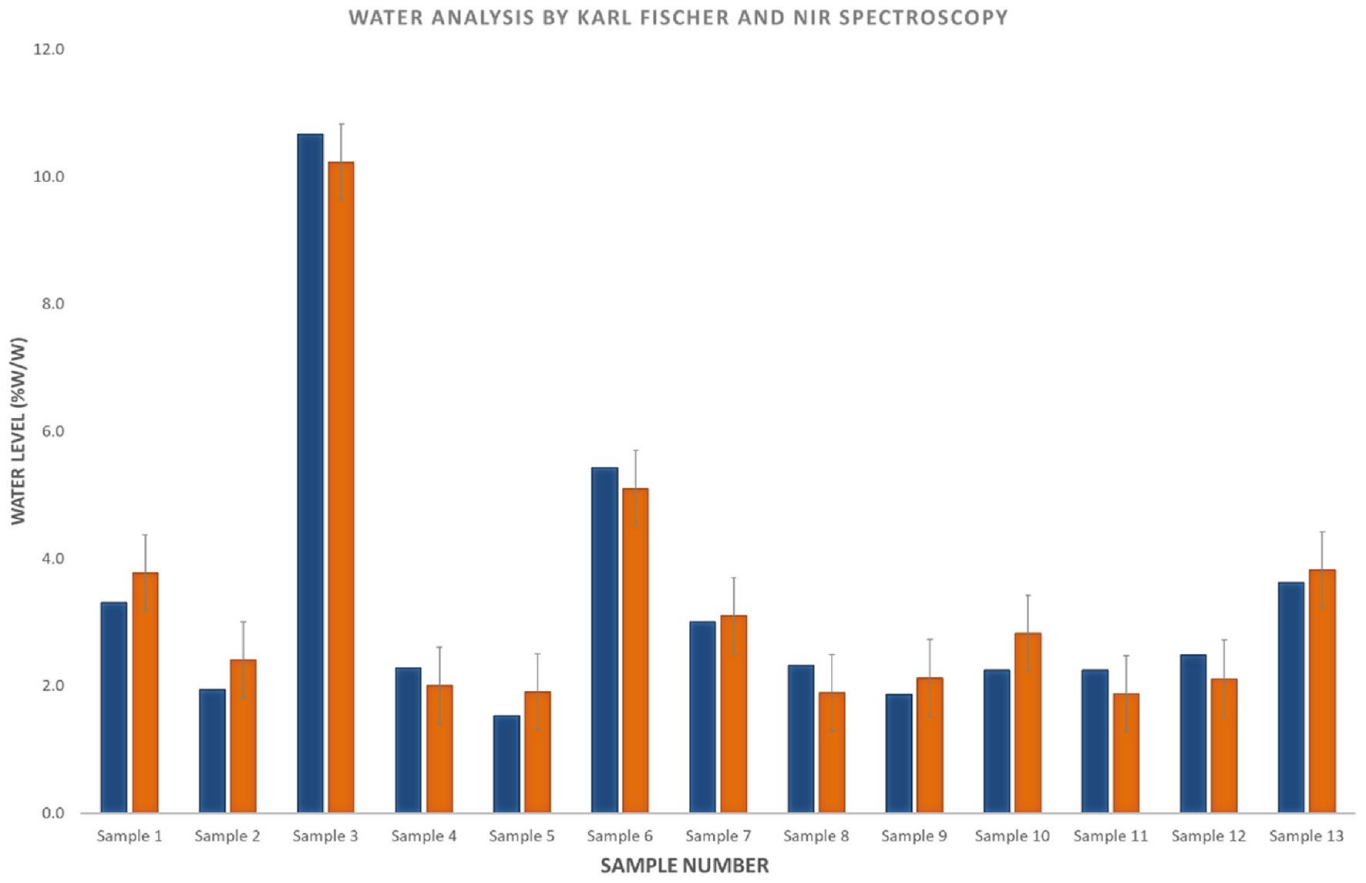
Comparison of water values measured by Karl-Fischer titration (blue) and predicted by NIR spectroscopy (orange). The Standard Error of Prediction associated with the NIR measurement for water (± 0.6% w/w) is indicated on the plot.

For water, the comparison of NIR-predicted and GC measured values was hindered by the LOQ of the GC-TCD method and the comparatively low water levels present in many of the e-liquid formulations. As a result, 38% of the samples were below the LOQ of the GC-TCD method, from which the data could not be compared with the NIR predicted values (Supplementary Fig. 2). Subsequently, a Karl-Fischer titration has been carried out on a subset of samples containing low amount of water (0–10% w/w) to assess the performance of the NIR analysis. The results have shown comparable results between the water content determined by Karl-Fischer titration and the NIR spectroscopy predicted values, taking into account the Standard Error of Prediction associated with the PLS model for water (0.6% w/w) (Fig. 4). The window for the prediction is indicated in Fig. 4, in the form of error bars. Although the range for the water content was 0–10% w/w, water ingress has been observed in all samples, due to the hydroscopic nature of both PG and VG.

Collectively, these results suggest that our NIR is more sensitive for predicting the levels of water in e-liquids than the GC-TCD method used.

## Discussion

The use of NIR spectroscopy combined with a robust chemometric model (PLS regression) has allowed the development of a method for the accurate prediction of concentrations of analytes, namely PG, VG, water and nicotine, in e-liquid formulations.

The results suggest that the NIR methodology is suitable for the rapid and simultaneous estimation of PG, VG, water and nicotine in e-liquids with acceptable confidence (R^2^ ≥ 0.995 for all calibrations and expended standard error of prediction of 1.84, 1.88, 0.62 and 0.22% w/w for PG, VG, water and nicotine, respectively). As such, it may be an appropriate and much quicker alternative to classical methods such as GC. The approach specifically allows the simultaneous prediction within minutes of the concentrations of the four analytes in e-liquids with minimal sample preparation. The NIR method was more sensitive than GC for water analysis. Once the calibration curves for the compounds of interest are established there is no need for additional standard preparations or system calibration prior to the sample analysis. Importantly, no sample preparation is needed for the analysis of e-liquid formulations (i.e., no use of extraction solvents, purification steps or reagents), allowing ease of use and limiting costs, preparation time and environmental impact (solvent). The NIR methodology would therefore be favoured for applications requiring fast analysis and high throughput, such as screening of key ingredients in e-liquids.

The NIR methodology has potential applications in product development (e.g., confirmation of formulation, product safety assessment), in manufacture (e.g., production checks, quality control) or to investigate potentially illicit goods. In this regard, the NIR spectrometer can be easily operated by non-specialists.

A potential limitation of the methodology may be the continuous expansion of variety in e-liquids, which may incorporate novel flavourings and/or additives that could interfere with the prediction of compounds of interest, especially nicotine. However, this limitation can be addressed by periodic review and update of the PLS models to reflect the diversity of e-liquid formulations in the market/product portfolio.

## Methods

### Materials

The e-liquids comprised PG (CAS #57-55-6, Merck), VG (CAS #56-81-5), water (CAS #7732-18-5, Merck), and nicotine (CAS #56-81-5, Nicobrand). All other solvents and chemicals were from Merck, including methanol (CAS #67-56-1), isopropanol (CAS #67-63-0), trans-anethole (CAS #4180-23-8), dry methanol, Hydranal Composite 5, and Hydranal water standard 10 mg/g.

### E-liquid samples

For the NIR calibration models, sets of e-liquids were formulated covering the following working range for each analyte: 30–70% w/w PG, 18–65% w/w VG, 0.6–18% w/w water and 0.2–6% w/w nicotine. To address other sources of variation, the calibration sample set contained both unflavoured and flavoured liquids, including tobacco, fruit, mint and complex flavourings (Supplementary Table 5) based on sample availability and commercial relevance. The following numbers of data points were used for each calibration model: PG, 136 (125 calibration, 11 validation); VG, 137 (123 calibration, 14 validation); water, 136 (124 calibration, 12 validation); nicotine, 268 (221 calibration, 47 validation samples).

To assess the performance of the NIR method for PG, VG, nicotine and water, a test set of 49 commercially available BAT e-liquids was analysed in parallel by GC and NIR spectroscopy. The test e-liquids contained different flavourings and had the following analyte ranges: 25–55% w/w PG, 35–65% w/w VG, 0.6–25% w/w water, and 0.2–6% w/w (3–56 mg/mL) nicotine (Supplementary Tables 6–9).

A set of 13 additional e-liquids was analysed by NIR spectroscopy and Karl-Fischer analysis to further assess performance of the water calibration. The analyte range was PG: 50% w/w, VG: 50% w/w, nicotine: 0–3% w/w, water: 0–10%.

### Near infrared spectroscopy

For NIR spectroscopy analysis, the e-liquid samples were introduced in 5 mm glass vials. All samples were analysed in duplicate, due to homogeneity of the samples, stability of the instrument and reproducibility of the spectra, on an ABB MB3600 FT-NIR (Fourier Transform—Near Infrared) spectrometer equipped with a temperature-controlled vial holder set at 30 °C. Spectrum acquisition to establish the calibrations was carried out via the HorizonMB version 3.4.0.3 software (working range, 3996–10,000 cm^−1^; resolution, 8 cm^−1^; 64 scans, gain 27.14). Calibration models were constructed by using the calibration wizard tool within HorizonMB.

Sample analysis to assess the performance of the method was carried out using the user software, Horizon Workplace version 2.2. All samples for the method performance assessment were analysed in triplicate.

### Partial least squares regression

The calibration models were generated by using PLS regression to establish a linear correlation between the NIR spectral data (*X*) and the levels of analyte present in the e-liquid sample (*Y*). Each model relies on the calculation of latent variables, also called “factors” ^21^. Projection of the spectral data onto an individual factor constitutes the scores of the sample on this factor. To establish the predictive model, the information provided by the spectrum can be captured in the form of multiple factors, leading to multiple scores. These factors (latent variables) are calculated to maximise the variability in the spectral data set that is correlated to the variable of interest. As such, the PLS factors have the same dimensions as a single spectrum and each factor should be orthogonal to the other PLS factors. Combinations of the PLS factors weighted with a set of PLS scores (one for each factor for each spectral sample) can reconstruct any specific calibration spectrum. The PLS scores can be regressed against the property/analyte of interest. The regression equation obtained can then be used to predict the property/analyte concentration for an unknown sample by reconstructing it from a combination of the same latent variable (factors) and newly calculated scores for this unknown sample.

Any number of PLS factors can be calculated for a particular data set within the limits set by the smallest dimension; however, the number of factors used in any model should be limited to the minimum number that fully characterizes the variability in the dataset. The performance of the model is related to the number of factors included: too few will underfit the model, leading to a high error of prediction; an excessive number of factors in a model will produce “over fitting”, whereby the algorithm is trying to model the “noise” within the dataset. The optimum number of factors for a particular model will be dependent on each data set and will increase with the number of sources of variance within that dataset ^22,23^.

The advantages of multivariate techniques, such as PLS, are that they can deconvolute complex data sets and establish a relationship (correlation) between the data set (spectra) and the sample properties (e.g., concentrations of analytes in solution). This can be used to predict multiple properties from a single spectrum of an unknown sample of the same type. In addition, multivariate techniques can also simultaneously calculate statistics (e.g., spectral residual, Mahalanobis distance, etc.) that may indicate whether the predictions obtained are reliable.

Here, the precision and optimization of the PLS regression-based calibration models were evaluated based on the highest value of *R*^2^ for both calibration and validation sets. In addition, the Standard Error of Prediction of the NIR results (SEP) was also estimated by calculating the root mean square of differences (RMSD) on the calibration set.

$$RMSD= \sqrt{\frac{{\sum_{1}^{n}({P}_{n}-{R}_{n})}^{2}}{N}}$$

where ${P}_{n}$ is the predicted value, ${R}_{n}$ is the reported (theoretical) value for sample *n*, and *N* is the total number of samples.

For applied work, the calculated values were subsequently multiplied by 2 to provide a confidence interval of 95.4% for the prediction. This value is referred to as “expanded SEP”.

### Gas chromatography analysis

Samples for GC analysis were prepared by extracting 0.1 g of e-liquid with 20 mL of extracting solution (7.5 mL of methanol + 1.0 mL of trans-anethole in a total volume of 5 L of isopropanol). The mixture was shaken for 45 min at 150 rpm before being transferred into 2 mL amber vials. A 1 mL aliquot of extracted sample was injected in split mode (5:1) into an Agilent 6890/7890 GC equipped with a flame ionisation detector (FID) and a thermal conductivity detector (TCD). For PG, VG and nicotine analysis, the conditions were DB-WAX column, 15 m × 0.53 mm × 1.0 mm; inlet temperature, 220 °C; FID temperature, 250 °C; hydrogen detector flow rate, 30 mL/min; column flow rate (helium), 10 mL/min. For water analysis, the following conditions were used: Poroplot Q column, 25 m × 0.53 mm × 20 mm; inlet temperature, 220 °C; TCD temperature, 220 °C; column flow rate (helium), 30 mL/min. For all analytes, the oven temperature was initially set at 105 °C, increased at a rate of 10 °C/min to 180 °C, and then increased at a rate of 5 °C/min to 195 °C. Agilent OpenLab 2.6 software was used to acquire and process the data.

### Karl-Fischer analysis

Moisture content was also determined by Karl-Fischer analysis. Each e-liquid sample was weighed into a conical flask and 50 mL of dry methanol was added. The flask was closed with a stopper and sealed with parafilm. The sample was shaken at 155 rpm for 30 min and a 5 mL aliquot was titrated against Hydranal Composite 5 using a calibrated Mettler Toledo Karl-Fischer V30 Volumetric Titrator. The samples were analysed in triplicate.

### Data analysis

The calibration models for the NIR analysis of samples were developed with the HorizonMB software (version 3.4.0.3) using the in-built calibration wizard.

Pre-processing was carried out with the software’s wizard using a Norris gap algorithm of the second order (gap length: 30 for PG, 35 for VG, 25 for water, 50 for nicotine).

The PLS report for each calibration was subsequently exported to Microsoft Excel. The results of the NIR and GC analyses were entered into Excel and the values were compared by linear regression.

### Supplementary Information

Supplementary Information.

## Data Availability

The data generated and analysed during this study are included in the published article and its supplementary information file. Calibration files can be provided by corresponding author on reasonable request.
