# Supplementary material for: Rapid determination of levels of the main constituents in e-liquids by near infrared spectroscopy
Source: Sci Rep. 2023 Aug 19;13:13501. doi: 10.1038/s41598-023-40422-z (PMC10439909; doi:10.1038/s41598-023-40422-z)
Supplement: Supplementary file 1 — Supplementary Information. [file 41598_2023_40422_MOESM1_ESM.pdf]

Supplementary Information

Near infrared spectroscopy: a tool for rapid determination of levels of the main constituents in e-liquids

Anaïs R. F. Hoffmann, Jana Jeffery, Paul Dallin, John Andrews, Michał Brokl

Supplementary Table 1. Results of the factor analysis for PG, computed by the HorizonMB software.

Factor Analysis for PG				
Factor	R^2	F-Ratio	SEC	RMSEC
1	0.99161	3.4059	1.192872	1.192887
2	0.994951	2.049286	0.929088	0.929089

Supplementary Table 2. Results of the factor analysis for VG, computed by the HorizonMB software.

Factor Analysis for VG				
Factor	R^2	F-Ratio	SEC	RMSEC
1	0.930458	45.14719	3.510784	3.510785
2	0.994896	3.313719	0.9551	0.9551

Supplementary Table 3. Results of the factor analysis for nicotine, computed by the HorizonMB software.

Factor Analysis for nicotine				
Factor	R^2	F-Ratio	SEC	RMSEC
1	0.41769	120.3343	1.175867	1.175869
2	0.530772	96.96378	1.057944	1.057945
3	0.827341	35.6757	0.643192	0.643195
4	0.931091	14.23696	0.407256	0.407257
5	0.949274	10.48302	0.350275	0.350276
6	0.97175	5.839663	0.262024	0.262044
7	0.976387	4.882003	0.24014	0.240157
8	0.993703	1.301701	0.1243	0.124301
9	0.995161	1	0.109202	0.109206

Supplementary Table 4. Results of the factor analysis for water, computed by the HorizonMB software.

**Factor Analysis for nicotine**				
**Factor**	**R^2**	**F-Ratio**	**SEC**	**RMSEC**
1	0.984096	6.397774	0.79002	0.790021
2	0.994928	2.040403	0.447991	0.447991
3	0.996973	1.217666	0.347517	0.347518
4	0.997495	1.007919	0.3175	0.3175

Supplementary Table 5. E-liquid distribution across flavours and nicotine content used to establish a PLS model.

Nicotine level	Fruit	Mint	Other	Tobacco	Unflavoured	Vanilla	Grand Total
Zero (0 % w/w)	24	2	2	4	6		38
Med (0.6-1.6 % w/w)	12	2	4	8	40	6	72
Low (<0.6% w/w)	19	2		14	22	2	59
High (>1.6 % w/w)	39	14	6	8	28	4	99
**Grand Total**	**94**	**20**	**12**	**34**	**96**	**12**	**268**

Supplementary Table 6. E-liquid distribution across flavours and PG content used for the performance evaluation of the method

**PG content**	**Fruit**	**Mint**	**Other**	**Tobacco**	**Vanilla**	**Grand Total**
Low (<30% w/w)	2					2
Med (30-60% w/w)	19	10	4	12	2	47
**Grand Total**	**21**	**10**	**4**	**12**	**2**	**49**

Supplementary Table 7. E-liquid distribution across flavours and VG content used for the performance evaluation of the method

**VG content**	**Fruit**	**Mint**	**Other**	**Tobacco**	**Vanilla**	**Grand Total**
High (.60% w/w)	2					2
Med (30-60 % w/w)	19	10	4	12	2	47
**Grand Total**	**21**	**10**	**4**	**12**	**2**	**49**

Supplementary Table 8. E-liquid distribution across flavours and nicotine content used for the performance evaluation of the method

**Nicotine content**	**Fruit**	**Mint**	**Other**	**Tobacco**	**Vanilla**	**Grand Total**
High (>1.6% w/w)	6	4		4		14
Low (<0.6% w/w)	6	3	2	3		14
Med (0.6 – 1.6% w/w)	8	3	2	4	1	18
Zero (0% w/w)	1			1	1	3
**Grand Total**	**21**	**10**	**4**	**12**	**2**	**49**

Supplementary Table 9. E-liquid distribution across flavours and water content used for the performance evaluation of the method

**Water content**	**Fruit**	**Mint**	**Other**	**Tobacco**	**Vanilla**	**Grand Total**
High (>10% w/w)	6	2	1	3	1	13
Low (<3% w/w)	4	1		2		7
Med (3-10% w/w)	5	5	2	5		17
Zero (0 % w/w_	6	2	1	2	1	12
**Grand Total**	**21**	**10**	**4**	**12**	**2**	**49**

Supplementary Table 10. Comparison between predicted values by NIR spectroscopy and measured values by GC-FID for PG for the method performance evaluation

Sample ID	NIR - PG (% w/w)		GC - PG (% w/w)		PG recoveries (GC/NIR)
	Mean	Standard deviation	Mean	Standard deviation	
Sample_1	54.7	0.1	52.2	0.1	95%
Sample_2	54.5	0.2	51.9	0.1	95%
Sample_3	54.1	0.1	50.0	0.1	92%
Sample_4	50.9	0.1	49.3	0.1	97%
Sample_5	51.3	0.2	49.5	0.3	96%
Sample_6	52.5	0.1	49.1	0.4	94%
Sample_7	52.8	0.1	49.5	0.3	94%
Sample_8	53.3	0.2	52.2	0.1	98%
Sample_9	54.5	0.1	52.1	0.0	96%
Sample_10	52.6	0.0	49.9	0.3	95%
Sample_11	54.4	0.1	51.6	0.2	95%
Sample_12	53.1	0.0	49.1	0.1	93%
Sample_13	53.2	0.2	51.2	0.1	96%
Sample_14	54.6	0.2	51.2	0.1	94%
Sample_15	52.5	0.2	47.7	0.3	91%
Sample_16	54.0	0.1	50.3	0.3	93%
Sample_17	54.4	0.2	53.1	0.1	98%
Sample_18	52.9	0.1	52.4	0.1	99%
Sample_19	52.1	0.0	49.4	0.2	95%
Sample_20	51.3	0.1	49.8	0.1	97%
Sample_21	53.8	0.1	50.6	0.0	94%
Sample_22	54.0	0.4	53.2	0.2	99%
Sample_23	54.6	0.1	50.9	0.1	93%
Sample_24	32.3	0.0	31.0	0.1	96%
Sample_25	32.3	0.0	31.0	0.1	96%
Sample_26	32.0	0.1	30.6	0.1	96%
Sample_27	28.7	0.0	27.3	0.1	95%
Sample_28	29.5	0.0	28.0	0.1	95%
Sample_29	32.5	0.1	32.2	0.3	99%
Sample_30	33.1	0.1	32.0	0.1	97%
Sample_31	51.0	0.1	49.2	0.1	97%
Sample_32	51.1	0.1	45.8	0.2	90%
Sample_33	51.2	0.1	48.8	0.1	95%
Sample_34	48.0	0.1	46.1	0.1	96%
Sample_35	49.4	0.2	47.7	0.1	97%
Sample_36	49.6	0.1	49.0	0.2	99%
Sample_37	49.8	0.1	48.2	0.0	97%
Sample_38	51.2	0.1	48.2	0.1	94%
Sample_39	50.1	0.1	48.1	0.1	96%
Sample_40	51.5	0.0	48.4	0.1	94%
Sample_41	48.8	0.1	46.0	0.1	94%
Sample_42	49.4	0.2	48.2	0.1	97%
Sample_43	49.8	0.2	47.9	0.1	96%
Sample_44	48.7	0.2	46.4	0.2	95%
Sample_45	47.7	0.2	46.3	0.1	97%
Sample_46	49.4	0.1	43.4	0.1	88%

Sample ID	NIR - PG (% w/w)		GC - PG (% w/w)		PG recoveries (GC/NIR)
	Mean	Standard deviation	Mean	Standard deviation	
Sample_47	51.3	0.2	48.5	0.2	94%
Sample_48	50.3	0.1	47.9	0.1	95%
Sample_49	50.9	0.1	44.5	0.1	87%

Supplementary Table 11. Comparison between predicted values by NIR spectroscopy and measured values by GC-FID for VG for the method performance evaluation

Sample ID	NIR - VG (% w/w)		GC - VG (% w/w)		VG recoveries (GC/NIR)
	Mean	Standard deviation	Mean	Standard deviation	
Sample_1	34.4	0.0	33.0	0.1	96%
Sample_2	34.8	0.0	33.9	0.1	97%
Sample_3	34.9	0.0	32.6	0.1	93%
Sample_4	40.0	0.0	35.0	0.2	87%
Sample_5	39.5	0.0	34.6	0.3	88%
Sample_6	38.2	0.0	33.5	0.3	88%
Sample_7	37.6	0.0	32.7	0.3	87%
Sample_8	36.5	0.0	35.1	0.1	96%
Sample_9	34.9	0.1	33.6	0.1	96%
Sample_10	37.5	0.0	34.2	0.1	91%
Sample_11	34.7	0.1	32.5	0.2	94%
Sample_12	36.8	0.0	32.5	0.1	88%
Sample_13	36.4	0.1	34.9	0.1	96%
Sample_14	34.7	0.1	33.3	0.2	96%
Sample_15	38.5	0.0	33.3	0.2	87%
Sample_16	36.1	0.1	33.6	0.3	93%
Sample_17	34.9	0.1	34.7	0.2	99%
Sample_18	36.6	0.0	36.0	0.1	98%
Sample_19	34.9	0.0	34.6	0.2	99%
Sample_20	39.6	0.0	35.3	0.1	89%
Sample_21	36.7	0.0	34.6	0.1	94%
Sample_22	35.5	0.0	35.3	0.2	99%
Sample_23	34.6	0.0	34.0	0.1	98%
Sample_24	49.9	0.1	49.7	0.2	100%
Sample_25	49.7	0.2	49.7	0.2	100%
Sample_26	50.4	0.0	50.0	0.1	99%
Sample_27	61.7	0.1	60.9	0.1	99%
Sample_28	60.8	0.1	60.0	0.3	99%
Sample_29	50.4	0.1	50.5	0.5	100%
Sample_30	49.5	0.0	49.6	0.0	100%
Sample_31	48.2	0.1	50.2	0.0	104%
Sample_32	42.9	0.0	46.1	0.4	107%
Sample_33	47.7	0.0	48.8	0.1	102%
Sample_34	51.1	0.1	52.6	0.0	103%
Sample_35	49.9	0.1	50.3	0.0	101%
Sample_36	49.8	0.1	51.0	0.0	102%
Sample_37	49.4	0.1	50.7	0.0	103%
Sample_38	47.9	0.1	48.8	0.0	102%
Sample_39	49.4	0.2	50.6	0.0	102%
Sample_40	47.7	0.1	48.7	0.0	102%
Sample_41	51.1	0.1	50.1	0.1	98%
Sample_42	50.4	0.3	50.7	0.0	101%
Sample_43	49.4	0.1	50.1	0.0	101%
Sample_44	51.7	0.1	49.7	0.0	96%
Sample_45	52.9	0.2	50.6	0.0	96%
Sample_46	46.0	0.1	45.8	0.2	100%

Sample ID	NIR - VG (% w/w)		GC - VG (% w/w)		VG recoveries (GC/NIR)
	Mean	Standard deviation	Mean	Standard deviation	
Sample_47	47.6	0.1	48.4	0.0	102%
Sample_48	48.6	0.1	49.4	0.0	102%
Sample_49	43.3	0.0	45.0	0.4	104%

Supplementary Table 12. Comparison between predicted values by NIR spectroscopy and measured values by GC-FID for nicotine for the method performance evaluation

Sample ID	NIR Nicotine (% w/w)		GC - Nicotine (% w/w)		Nicotine recoveries (GC/NIR)
	Mean	Standard deviation	Mean	Standard deviation	
Sample_1	1.6	0.0	1.5	0.0	96%
Sample_2	1.1	0.0	1.1	0.0	99%
Sample_3	1.8	0.1	1.9	0.0	106%
Sample_4	0.1	0.0	<LOQ	N/A	N/A
Sample_5	0.4	0.0	0.3	0.0	80%
Sample_6	1.3	0.0	1.1	0.0	86%
Sample_7	1.9	0.0	1.7	0.0	91%
Sample_8	0.2	0.0	<LOQ	N/A	0%
Sample_9	1.1	0.0	1.0	0.0	97%
Sample_10	0.7	0.0	0.6	0.0	86%
Sample_11	1.7	0.0	1.5	0.0	93%
Sample_12	1.7	0.1	1.7	0.0	97%
Sample_13	0.1	0.0	<LOQ	N/A	N/A
Sample_14	1.3	0.0	1.1	0.0	88%
Sample_15	0.8	0.0	1.1	0.0	139%
Sample_16	1.4	0.1	1.2	0.0	92%
Sample_17	1.2	0.0	1.1	0.0	96%
Sample_18	0.0	0.0	<LOQ	N/A	N/A
Sample_19	1.1	0.1	1.2	0.0	104%
Sample_20	0.6	0.0	0.6	0.0	96%
Sample_21	1.8	0.0	1.7	0.0	99%
Sample_22	0.5	0.1	0.6	0.0	117%
Sample_23	1.7	0.0	1.8	0.0	110%
Sample_24	0.6	0.0	0.5	0.0	89%
Sample_25	0.5	0.0	0.5	0.0	99%
Sample_26	0.5	0.1	0.5	0.0	101%
Sample_27	0.3	0.1	0.2	0.0	92%
Sample_28	1.0	0.0	1.0	0.0	93%
Sample_29	0.0	0.0	<LOQ	N/A	100%
Sample_30	0.4	0.0	0.5	0.0	116%
Sample_31	1.2	0.1	1.1	0.0	89%
Sample_32	4.9	0.1	5.0	0.0	102%
Sample_33	1.6	0.0	1.57	0.0	95%
Sample_34	1.6	0.1	1.45	0.0	93%
Sample_35	1.0	0.0	0.55	0.0	55%
Sample_36	0.0	0.0	<LOQ	N/A	N/A
Sample_37	0.7	0.0	0.52	0.0	77%
Sample_38	1.7	0.0	1.54	0.0	93%
Sample_39	0.6	0.0	0.52	0.0	89%
Sample_40	1.7	0.0	1.59	0.0	96%
Sample_41	0.0	0.0	<LOQ	N/A	N/A
Sample_42	0.1	0.1	<LOQ	N/A	N/A
Sample_43	0.5	0.0	0.50	0.0	103%
Sample_44	1.2	0.0	1.03	0.0	84%
Sample_45	0.6	0.1	0.50	0.0	86%
Sample_46	5.0	0.0	4.95	0.0	100%

Sample ID	NIR Nicotine (% w/w)		GC - Nicotine (% w/w)		Nicotine recoveries (GC/NIR)
	Mean	Standard deviation	Mean	Standard deviation	
Sample_47	1.6	0.0	1.58	0.0	99%
Sample_48	1.1	0.1	1.04	0.0	99%
Sample_49	4.9	0.0	5.04	0.0	103%

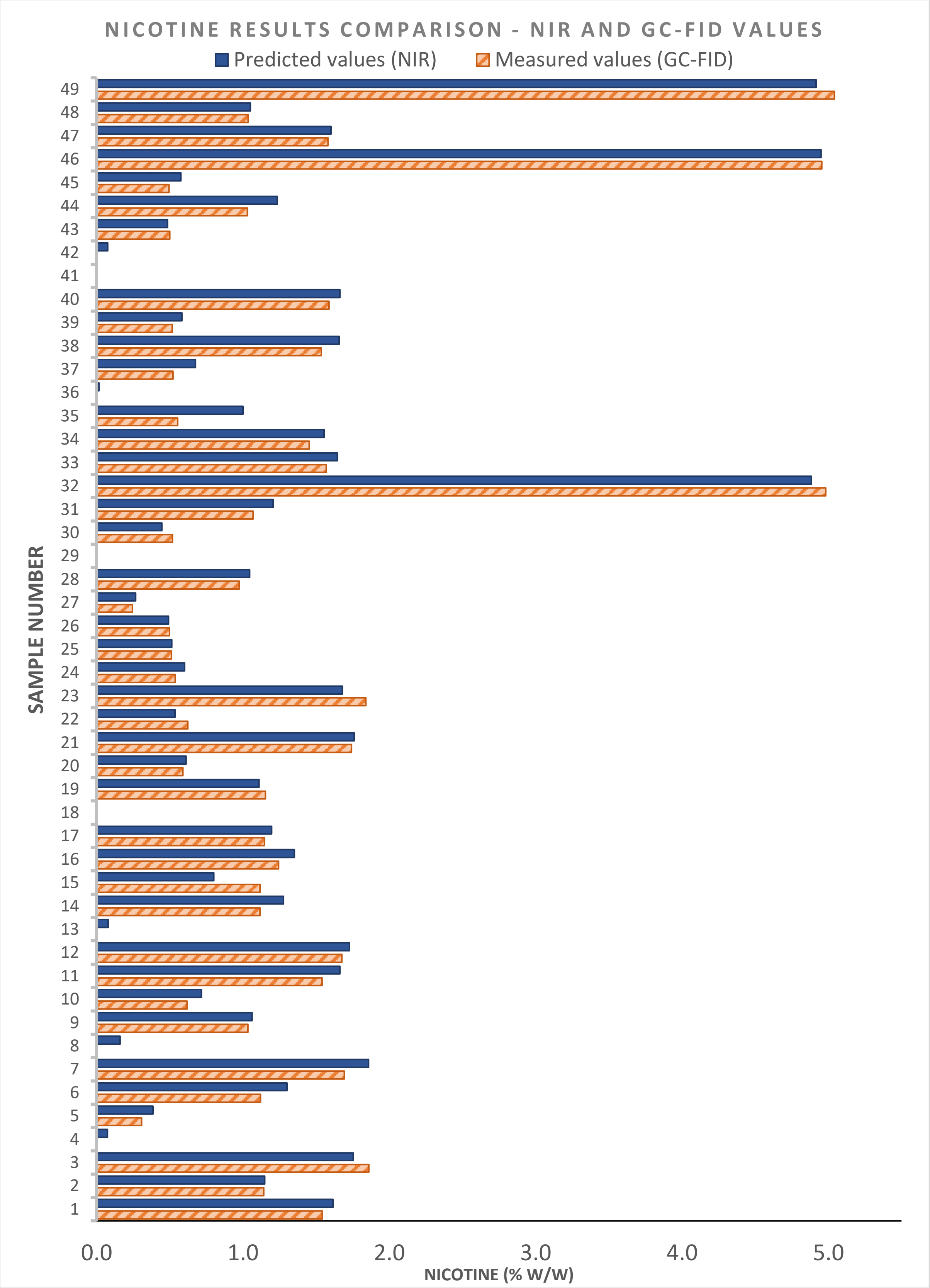

Supplementary Figure 1. Comparison of nicotine values predicted by NIR (blue) and those measured by GC-FID (orange) – cross validation analysis.

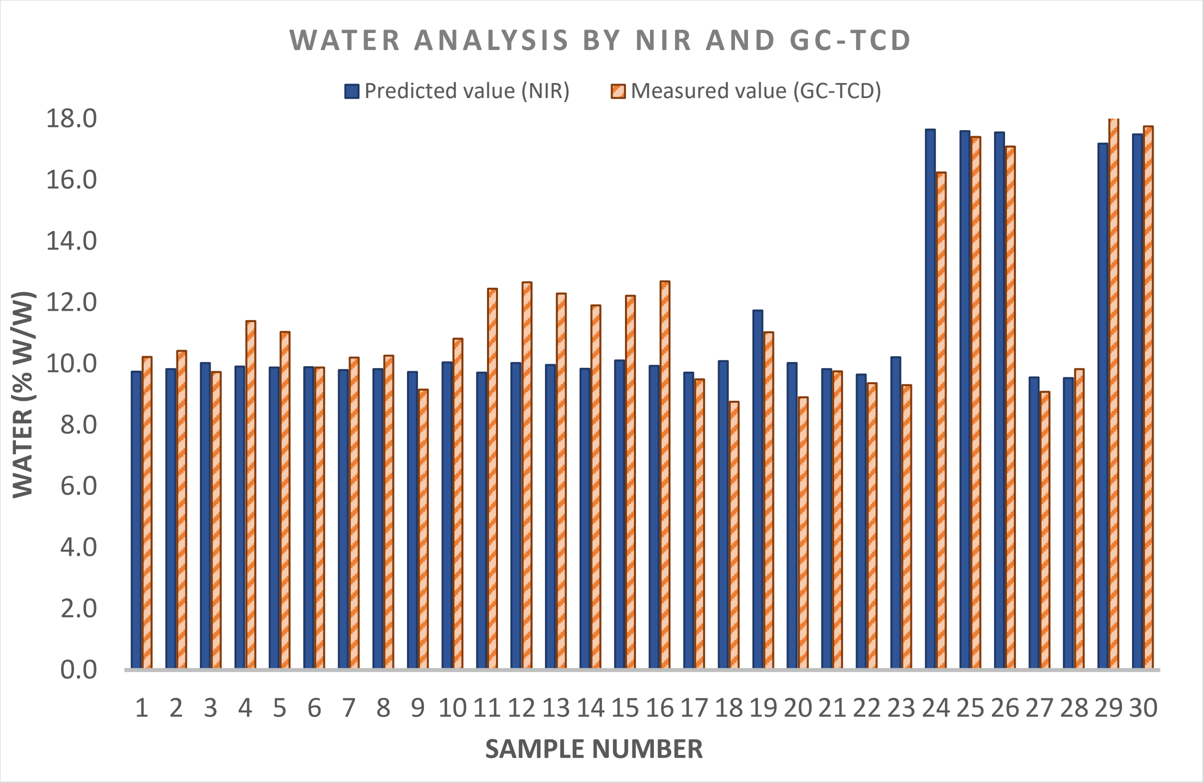

Supplementary Figure 2. Comparison of water values predicted by NIR (blue) and those measured by GC-TCD (orange) – cross validation analysis.
